# The first case of *Ochrobactrum intermedium* bacteremia in a pediatric patient with malignant tumor

**DOI:** 10.1186/s12879-021-06938-3

**Published:** 2021-12-14

**Authors:** Wenjing Wu, Yongmei Jiang, Wei Zhou, Xingxin Liu, Linghan Kuang

**Affiliations:** 1 Department of Laboratory Medicine, West China Second University Hospital, Sichuan University, Chengdu, China; 2 Key Laboratory of Birth Defects and Related Diseases of Women and Children (Sichuan University), Ministry of Education, Chengdu, China

**Keywords:** *Ochrobactrum*, *Ochrobactrum intermedium*, Bacteremia, Pediatric patient

## Abstract

**Background:**

*Ochrobactrum* spp. are non-fermenting, Gram-negative bacilli that are regarded as emerging human pathogens of low virulence that can cause infections. The first identified case of *Ochrobactrum intermedium* was reported in 1998 in a liver transplantation patient with liver abcess. There are no reports of infections in pediatric patients. Here, we report the first case of *O. intermedium* bacteremia in a pediatric patient.

**Case presentation:**

A two and a half years old male was admitted with fever, chills and nausea. He had been diagnosed as pineoblastoma and underwent surgical resection and chemotherapy. *O. intermedium* was isolated from his blood cultures and identified by matrix-assisted laser desorption/ionization time-of-flight mass spectrometry (MALDI-TOF MS), however, the Vitek II automated system failed to identify the organism. Then the pathogen was confirmed by 16S rDNA sequencing and average nucleotide identity result (ANI) confirmed the precise identification of *O. intermedium* at genomic level. In addition, the patient recovered well after antibiotic combined therapy.

**Conclusions:**

This, to our knowledge, is the first case of *O. intermedium* bacteremia in a pediatric patient with malignant tumor. Traditional biochemical identification methods such as API 20NE or VITEK2 system cannot differentiate *O. anthropi* and *O. intermedium*. MALDI-TOF may be a promising tool for rapid identification of microorganisms such as *O. intermedium*.

## Background

*Ochrobactrum* spp. belongs to the family *Brucellaceae* and it is widely distributed in the environment and clinical unit, such as soil, plant, water and indwelling catheters [1]. To date, there are 18 *Ochrobactrum* species with validly published names but only five of them, *O. anthropi*, *O. intermedium*, *O. haematophilum, O. pseudogrignonense* and *O. pseudintermedium*, were isolated from clinical specimens [2]. *O. anthropi* is the most common species recovered from clinical samples, and a variety of infections, such as catheter-related bloodstream infection, peritonitis or endophthalmitis, could be caused by this pathogen [3]. Unlike *O. anthropi, O. intermedium* infections were rare and up to now, only nine cases of *O. intermedium* infection were reported, and they usually cause infections in adult patients with bacteremia, prostatic abscess, endophthalmitis and infective endocarditis [2]. Here, we report the first case of *O. intermedium* bacteremia in a pediatric patient with malignant tumor.

## Case presentation

**Fig. 1:**
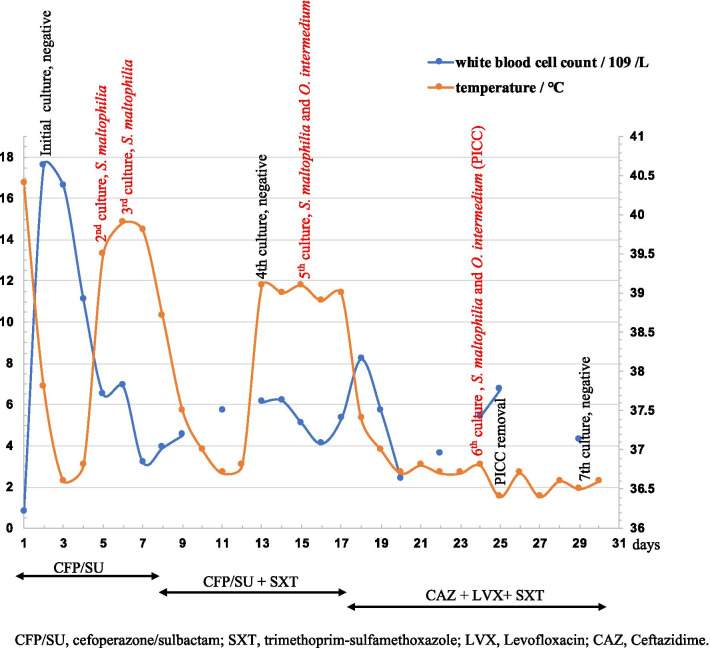
Temperature, white blood cell count and the therapeutic schedule of the patient

A two and a half years old male with fever was admitted to our hospital on August 2019. He had been diagnosed as pineoblastoma 7 months ago and underwent surgical resection and chemotherapy. He received a ventriculo-peritoneal shunt for obstructive hydrocephalus 4 months ago. He presented with fever (up to 40.4 °C), chills and nausea 2 days before hospital admission. Physical examination did not reveal any significant findings except pharyngeal hyperemia and swollen tonsils. His breath and heart sounds were normal. No redness or swelling occurred at the peripherally inserted central catheter (PICC) site, which had been in indwelled for 6 months, and the catheter was flushed. Computed tomography, urine examination and cerebrospinal fluid were normal. His white blood cell count and C-reaction protein concentration were 17.6 × 10^9^/L (96.3% neutrophils) and 130.5 mg/L (Fig. 1). Three sets of blood cultures were collected from peripheral venipuncture at the beginning of the febrile period, then he was treated with cefoperazone/sulbactam. Two of the blood cultures were positive and the pathogens were identified as *Stenotrophomonas maltophilia* by MALDI-TOF MS (bioMérieux, Marcy-l’Étoile, France). Susceptibility testing showed the organism was susceptible to sulfamethoxazole-trimethoprim, minocycline, ceftazidime, levofloxacin, chloramphenicol, and ticarcillin/clavulanate. The antibiotic treatment was changed to the combination of cefoperazone/sulbactam and sulfamethoxazole–trimethoprim. Then the boy remitted and the body temperature dropped to normal range within 3 days since the treatment was adjusted. The white blood cell counts also returned to normal level and repeated blood cultures (collected from peripheral vein) were negative. However, on the 13th day, his temperature increased again with the highest point of 39.1 °C, and blood specimens were collected from two peripheral veinpuncture sites (left hand and right foot). After 12 h of incubation, gram-negative bacilli were detected from one blood culture, which was collected from right foot. Two types of colonies grew in the blood agar after cultivation, which were identified as *S. maltophilia* and *O. intermedium* (strain 045999) by MALDI-TOF MS with 99.9% confidence; however, the Vitek II automated system (bioMérieux) failed to identify strain 045999. Genomic DNA of strain 045999 was prepared using the QIAamp DNA mini kit (Qiagen; Hilden, Germany) and were subjected to whole genome sequencing using the HiSeq X10 Sequencer (Illumina; San Diego, CA, USA). For the whole genome sequence of strain 045999, 5,619,077 reads and 1.69 GB bases were generated (coverage, $\times$400), which were assembled into a 4.9 Mb draft genome contains 82 contigs ≥ 200 bp in length (N*50*, 454,256 bp) with a 57.71 mol% GC content. ANI between strain 045999 and the type strain LMG 3301^T^ of *O. intermedium* (ACQA00000000) was determined using the JSpecies [4]. The ANI value was 97.57%, which was above the cutoff (≥ 95–96% ANI) defining the boundaries of bacterial species [5], confirming that the identification was precise. The same organisms were isolated from the other blood culture collected from left hand after 4 days incubation. Antimicrobial susceptibility testing was performed using broth microdilution with the Vitek II automated system (bioMérieux) and interpreted according to the guidelines for other non-Enterobacterales in CLSI M100 (2019). The strain 045999 was susceptible to sulfamethoxazole–trimethoprim, minocycline, tigecycline, cefepime, levofloxacin, meropenem, imipenem (Table 1). The strain 045999 formed beige, translucent, shiny and mucoid colonies on blood agar within 24 h of incubation at 35 °C and gram-staining indicated gram-negative, rod-shaped bacteria without spores. The antimicrobial susceptibility of the *S. maltophilia* was consistent with previous results. According to the results of susceptibility test, the *S. maltophilia* was susceptible to levofloxacin and ceftazidime, and strain 045999 was also susceptible to sulfamethoxazole–trimethoprim and levofloxacin. Therefore, cefoperazone/sulbactam was replaced with levofloxacin and ceftazidime, and sulfamethoxazole–trimethoprim was continued as before. One week after the treatment, blood cultures were obtained from both peripheral venipuncture and PICC. The blood culture obtained from PICC was positive with the growth of *S. maltophilia* and *O. intermedium* whereas the blood obtained from peripheral venipuncture was negative. Meanwhile, the PICC was removed and the culture was negative. Therefore, the therapy mentioned above was continued for 10 days, and the patient discharged after recovery.

**Table 1:** Antimicrobial susceptibility of the strain 045999

Antimicrobial	MIC (mg/L)	Category	Antimicrobial	MIC (mg/L)	Category
Amikacin	64	R	Imipenem	1	S
Aztreonam	≥ 64	R	Levofloxacin	0.25	S
Cefepime	4	S	Meropenem	≤ 0.25	S
Cefoperazone/sulbactam^a^	32	I	Minocycline	≤ 1	S
Ceftazidime	32	R	Piperacillin/tazobactam	≥ 128	R
Ciprofloxacin	≤ 0.25	S	Tigecycline	0.5	S
Colistin	64	R	Tobramycin	≥ 16	R
Gentamicin	8	I	Sulfamethoxazole–trimethoprim	≤ 1/19	S

*S* susceptible, *I* intermediate, *R* resistant

^a^The breakpoint of cefoperazone instead of cefoperazone/sulbactam

## Discussion and conclusion

*Ochrobactrum* spp. non-fermenting, Gram-negative bacilli are regarded as emerging human pathogens of low virulence that can cause infections not only in immunocompromised patients but also in human without underlying diseases [6]. Unlike *O. anthropi*, infection caused by *O. intermedium* is relatively rare. The first identified case of *O. intermedium* was reported in 1998 in a post-liver transplantation patient with a liver abcess, however, the microorganism was wrongly identified as *O. anthropi* by biochemical testing, and it was confirmed to be *O. intermedium* by PCR amplification of 16 S rDNA gene [7]. But up to now, there were few reports about the *O. intermedium* infection. Based on our literature search in PubMed, only nine cases of *O. intermedium* infection were reported (Table 2). In the reported cases, *O. intermedium* causes various infections in immunocompromised patients, such as liver abscess, prostatic abscess, and infective endocarditis [7–9]. Moreover, there were two cases reporting that *O. intermedium* were responsible for causing endophthalmitis and pelvic abscess in immunocompetent hosts [10, 11]. In two cases, *O. intermedium* was isolated from fecal in patients suffering from either bacteremia or bacteremia associated with liver abscess, indicating that the gastrointestinal tract was possibly the source of infection [7, 12]. Of the nine cases, seven patients male (so do our case), five patients had bacteremia, two of whom were associated with catheters [2, 13]. *O. anthropi* infection is usually seen in immunocompromised patients with indwelling catheters, and catheter-related bacteremia is also most reported in immunocompromised children, especially among boys under 10 years old with malignancies [6, 14]. In the present case, similar to the *O. anthropi* bacteremia, *O. intermedium* bacteremia occurred in pediatric patient with PICC, who underwent chemotherapy and was immunocompromised. Compared to *O. anthropi, O. intermedium* infection is rare, and it is likely that some infections thought to be caused by *O. anthropi* may be actually caused by *O. intermedium*, since these species cannot be differentiated by traditional biochemical identification methods such as API 20NE or VITEK2 system leading to misidentification. Thus, the incidence of *O. intermedium* infection in human may be underestimated [6, 15]. In addition, *O. intermedium* may undergo an ecological niche specialization and some lineages may shift toward an adaption to the human host based on a phylogenomic approach [16]. In order to accurately identify *O. anthropi* and *O. intermedium*, molecular typing was recommended, however, it was more time-consuming and complicated compared with MALDI-TOF. In this case, the pathogen was initially identified as *O. intermedium* by MALDI-TOF, which was consistent with previous study [8, 13, 17]. ANI result confirmed the precise identification of *O. intermedium* at genomic level, indicating MALDI-TOF is a promising tool for rapid identification of *O. intermedium* in clinical situations, though more studies are required.

**Table 2:** Case reports of *O. intermedium* infection

Author/Year	Age (year)/sex	Diagnose	Underlying disease	Antibiotic regimen	Prognosis	References
Moller et al./1999	45/F	Liver abcess	Liver transplant	CIP/metronidazole	Improved	[7]
Apisarnthanarak et al./2005	74/M	Bacteraemia	Bladder cancer	IPM/CIP	Improved	[12]
Vaidya et al./2006	49/M	Pelvic abscess	Non	LVX/metronidazole	Improved	[11]
Dharne et al./2008	26/M	Non-ulcer dispepsia	Unknown	Unknown	Improved	[19]
Jacobs et al./2013	34/M	Endophthalmitis	Vitrectomy, lensectomy, removal of intraocular foreign body	Intravitreal MOX	Improved	[10]
Rodriguez-Villodres et al./2016	70/M	Prostatic abscess	Bladder cancer	CIP/cotrimoxazole	Improved	[8]
Bharucha et al./2016	23/M	Infective endocarditis	Renal failure	MEM/MIN	Improved	[9]
Hirai et al./2016	86/M	Bacteraemia	Cerebral infarction	MEM	Improved	[17]
Kassab et al./2021	84/F	Bacteraemia	Primary sclerosing cholangitis	MIN	Improved	[13]
Present case	2/M	Bacteraemia	Pineoblastoma	LVX/CAZ/SXT	Improved	–

*CIP* ciprofloxacin, *IPM* imipenem, *LVX* levofloxacin, *MOX* moxifloxacin, *MEM* meropenem, *MIN* minocycline, *CAZ* ceftazidime, *SXT* sulfamethoxazole–trimethoprim

*Ochrobactrum* isolates were highly resistant to β-lactams, due to expression of AmpC β-lactamase, but they were mostly susceptible to levofloxacin, imipenem, ciprofloxacin, TMP-SMX and meropenem [6]. Susceptibility to colistin seems to be a potential marker for distinguishing between *O. anthropi* and *O. intermedium*, as *O. anthropi* is usually sensitive, whereas *O. intermedium* is usually resistant to this class of drugs [6]. In present case, *S. maltophilia* was isolated along with *O. intermedium*, indicating that it can cause polymicrobial infections in bloodstream infection (BSI), but *O. anthropi* seldom cause polymicrobial infections with other bacteria in BSI. Thus, the difficulty of treatment was increased. Catheter-related BSI was highly suspected in this case, but it was denied, because the blood culture obtained from PICC was positive, however, blood culture obtained from peripheral venipuncture and catheter culture were both negative. This is likely due to the use of antibiotics, therefore, the correct collection location, timing or times of blood sample play important roles in diagnosis of BSI infections [18]. Although both the two isolates were susceptible to sulfamethoxazole–trimethoprim, the combination treatment (levofloxacin, ceftazidime and sulfamethoxazole–trimethoprim) was used in our case and showed a good response.

In conclusion, here we reported the first case of *O. intermedium* bacteremia in a pediatric patient with malignant tumor. Infection caused by *O. intermedium* is uncommon, however, the incidence is probably being underestimated due to the difficult identification, and the clinical significance of *O. intermedium* remains unclear. Currently, the identification of *O. intermedium* is confirmed by molecular biological technique, however, this method may not be available in many clinical laboratories. Instead, MALDI-TOF MS could be helpful in identifying *O. intermedium*.

## Data Availability

The draft genome sequence of strain 045999 has been deposited into GenBank/DDBJ/EMBL under Accession number JABXOG000000000.

## Abbreviations

MALDI-TOF MS: Matrix-assisted laser desorption/ionization time-of-flight mass spectrometry
ANI: Average nucleotide identity result
CLSI: Clinical Laboratory Standards Institute
PICC: Peripherally inserted central catheter
BSI: Bloodstream infection
